# Nomogram of intra-abdominal infection after surgery in patients with gastric cancer: A retrospective study

**DOI:** 10.3389/fonc.2022.982807

**Published:** 2022-10-03

**Authors:** Yue Zhang, Zhengfei Wang, Zarrin Basharat, Mengjun Hu, Wandong Hong, Xiangjian Chen

**Affiliations:** ^1^ Department of Otolaryngology, Wenzhou People’s Hospital, Wenzhou, China; ^2^ Department of Gastrointestinal Surgery, the First Affiliated Hospital of Wenzhou Medical University, Wenzhou, China; ^3^ Department of Hepato-biliary Surgery, The Quzhou Affiliated Hospital of Wenzhou Medical University, Quzhou People’s Hospital, Quzhou, China; ^4^ Jamil-ur-Rahman Center for Genome Research, Dr. Panjwani Center for Molecular Medicine and Drug Research, International Center for Chemical and Biological Sciences, University of Karachi, Karachi, Pakistan; ^5^ Department of Pathology, Zhuji Affiliated Hospital of Wenzhou Medical University, Shaoxing, China; ^6^ Department of Gastroenterology and Hepatology, the First Affiliated Hospital of Wenzhou Medical University, Wenzhou, China

**Keywords:** gastric cancer, surgery, postoperative complications, intra-abdominal infection, receiver operating characteristic curve, nomogram

## Abstract

**Background:**

Surgical resection is still the primary way to treat gastric cancer. Therefore, postoperative complications such as IAI (intra-abdominal infection) are major problems that front-line clinical workers should pay special attention to. This article was to build and validate IAI’s RF (regression function) model. Furthermore, it analyzed the prognosis in patients with IAI after surgery for stomach cancer. The above two points are our advantages, which were not involved in previous studies.

**Methods:**

The data of this study was divided into two parts, the training data set and the validation data set. The training data for this article were from the patients treated surgically with gastric cancer in our center from December 2015 to February 2017. We examined IAI’s morbidity, etiological characteristics, and prognosis in the training data set. Univariate and multivariate logistic regression analyses were used to screen risk factors, establish an RF model and create a nomogram. Data from January to March 2021 were used to validate the accuracy of the RF model.

**Results:**

The incidence of IAI was 7.2%. The independent risk factors for IAI were hypertension (Odds Ratio [OR] = 3.408, P = 0.001), history of abdominal surgery (OR = 2.609, P = 0.041), combined organ excision (OR = 4.123, P = 0.010), and operation time ≥240 min (OR = 3.091, P = 0.005). In the training data set and validation data set, the area under the ROC curve of IAI predicted by the RF model was 0.745 ± 0.048 (P<0.001) and 0.736 ± 0.069 (P=0.003), respectively. In addition, IAI significantly extended the length of hospital stay but had little impact on survival.

**Conclusions:**

Patients with hypertension, combined organ excision, a history of abdominal surgery, and a surgical duration of 240 min or more are prone to IAI, and the RF model may help to identify them.

## Introduction

Gastric cancer is one of the most prevalent malignancies worldwide. According to the 2020 global cancer data (1), gastric cancer ranks fifth and fourth in morbidity and mortality, respectively. With diagnostic techniques such as endoscopy, the detection ratio of non-advanced gastric cancer is increasing, especially in Japan and South Korea. However, in China, there is no nationwide screening for gastric cancer (2). Only a small percentage of patients with early stomach cancer could receive treatment with ESD (Endoscopic Submucosal Dissection) or EMR (Endoscopic mucosal resection) (3). The remaining patients with advanced stage were treated with subtotal gastrectomy/total gastrectomy and lymph node dissection. Despite significant advances in surgical and postoperative care techniques for gastric cancer, severe postoperative complications can still occur at a high rate and affect the prognosis of patients (4–6). Therefore, determining how to reduce the occurrence of IAI is critical. The analysis of risk factors and the establishment of prediction models have been widely used in clinical disease research. Eun Hye Kim et al. developed a valid predictive model that can be used to determine the patients who will receive non-curative ESD resection (7). Screening the risk factors of IAI after gastric cancer surgery and establishing a prediction model can help clinicians take targeted measures to prevent the occurrence of IAI. Many scholars have studied surgical site infections, including their incidence, risk factors, prognosis, etc. However, most were superficial incision infections. Research on deep infections is not comprehensive enough, such as modeling and validation. Our innovation lies in the inclusion of more risk factors that were not included in previous studies, such as PNI (Prognostic nutritional index), neoadjuvant chemotherapy, lesion location, surgical method, and pathological type, to establish an RF (regression function) model and verify its predictive value by internal validation. In addition to the risk factor analysis of IAI occurrence and the establishment of the RF model, this paper also verified the RF model, which was never seen in previous studies. Through multivariate logistic regression analysis, we can obtain this RF model, which can help us predict the probability of IAI for each patient. Finally, we also studied the impact of IAI on prognosis. This article added risk factors such as preoperative adjuvant chemotherapy. Since most previous authors have studied its relationship with overall postoperative complications (8, 9), this article aims to explore its relationship with a single complication, intraperitoneal infection, so this factor is included.

## Materials and methods

### Patients

**Figure 1:**
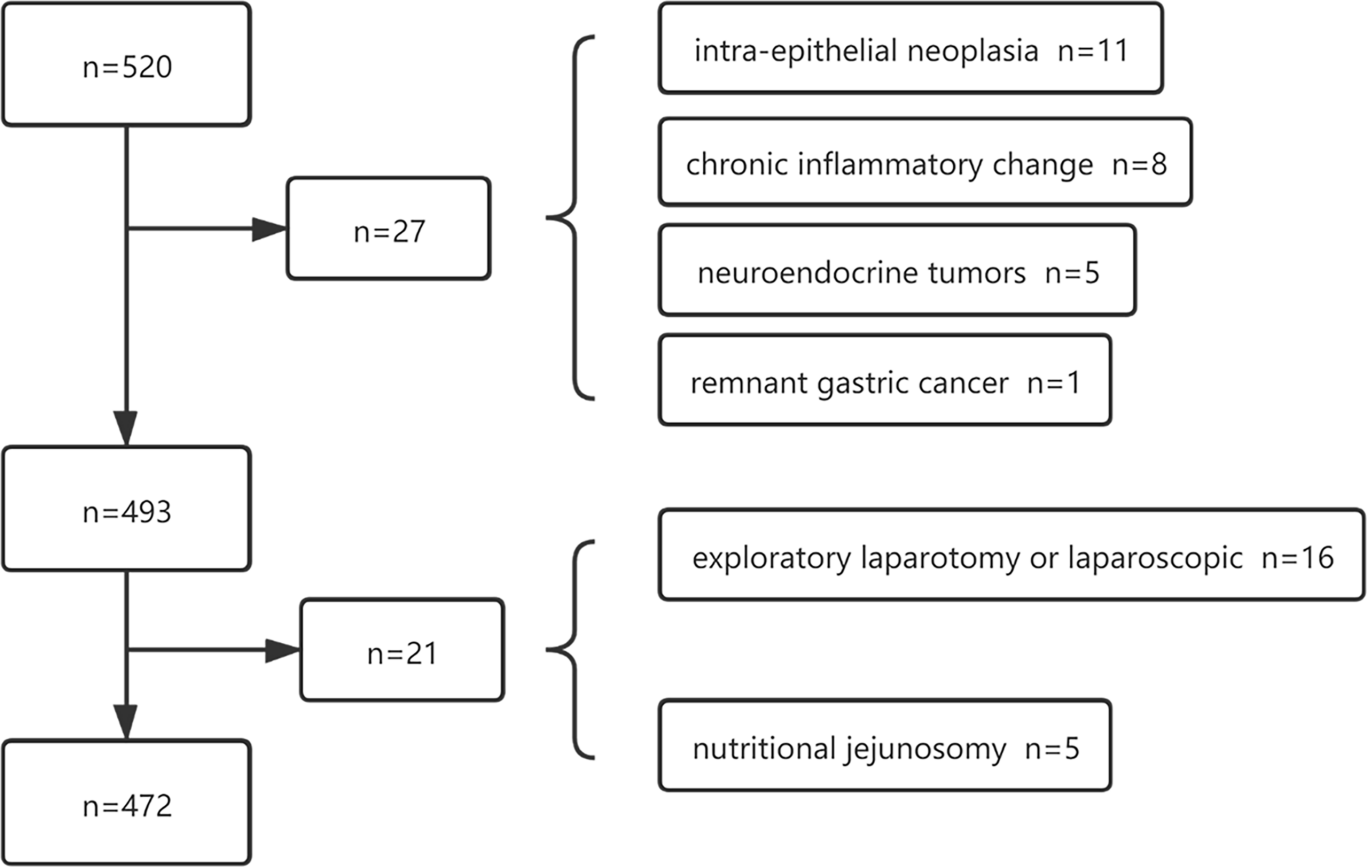
A total of 520 patients were initially screened and 472 patients were identified of the training data.

The paper collected the data from 520 gastric cancer patients who were admitted to the gastrointestinal surgery department of our hospital for surgery from December 2015 to February 2017. The inclusion criteria of this study were patients who were surgically treated with gastric cancer in our department, aged > 18 years old, and without organ dysfunction. The exclusion criteria had emergency surgery, postoperative pathologic indication of non-primary gastric cancer, extensive peritoneal metastasis without surgical treatment, and preoperative intra-abdominal infection. According to the pathological report, 27 out of 520 cases were classified as pathological inconsistencies, including 5 having a neuroendocrine tumor, 2 suffering from lymphoma, 8 with chronic inflammatory changes such as chronic ulcers, 11 having intra-epithelial neoplasia, 1 with remnant gastric cancer, and 21 cases excluded due to surgical inconsistencies. As is displayed in **Figure 1**, 472, patients who met the criteria were finally included in this study. Among the 472 patients, 413 underwent radical gastrectomy with D2 lymph node dissection, and 59 underwent palliative resection. In addition, 101 patients underwent laparoscopic-assisted radical gastrectomy. Based on the same inclusion and exclusion criteria, 135 patients were selected from January to March 2021 to validate the prediction model. Each tumor was pathologically diagnosed and staged according to the 8th edition of the AJCC (American Joint Committee on Cancer) TNM classification system of Gastric Cancer (10).

### Surgical procedures and perioperative management

The patients included in this article were treated by our experienced gastrointestinal surgery team. The scope of lymph node dissection and the mode of gastrointestinal reconstruction was determined according to the fourth edition of the Japanese gastric cancer treatment guidelines (11). According to the guidelines, patients in cT1a or cT1b without lymph nodes or distant metastases should undergo D1 or D1+ lymph node dissection. Standard D2 or D2+ lymphadenectomy is feasible for patients with the following requirements: T2-4 or N+. When a patient was preoperatively diagnosed as M1, it was decided whether to perform combined organ resection and enlarged lymph node radical resection to achieve the R0 resection standard. If radical surgery was impossible, palliative resection or gastrointestinal short-circuit surgery was performed to relieve the suffering of patients and improve their future quality of life. Preoperative patients were given routine fasting for 8h and intestinal cleaning. A postoperative drainage tube was routinely placed in the sub-hepatic and splenic fossa. Perioperative treatment with cephalosporins was routinely used to prevent infection until 3–5 days after surgery. If no drainage fluid is found or the drainage fluid is relatively clear for 2–3 days, and the patient has no discomfort such as abdominal pain/fever, the drainage tube may be removed. The antibiotic was changed based on bacterial susceptibility testing or clinical experience if a patient was diagnosed with IAI. The treatment of IAI included routine surgical monitoring and nursing, simple rubber tube drainage, double cannula flushing and drainage, analgesic, antipyretic, anti-infection treatment, maintenance of water & electrolyte balance, nutritional support, and surgery (12).

### Clinical and surgical outcomes

The following variables were obtained from the patient’s medical records at our hospital: Sex, age, BMI (Body Mass Index), chronic diseases (diabetes, hypertension), ASA (American Society of Anesthesiologists) score, preoperative chemotherapy history, earlier abdominal surgery, the existence of anemia/hypo-albuminemia, presence of hyperlipemia, site of primary carcinoma (clinical stage), time of operation, operation method, combined organ excision, PNI (Prognostic nutritional index), and BTF (perioperative blood transfusion history). The following formula counted PNI: 10 × serum albumin value (g/dL) + 0.005 × total lymphocyte count in the peripheral blood (per mm3) (13). BTF is a transfusion of red blood cells during hospitalization (14). The percentage of deaths occurring within 30 days of surgery is known as postoperative mortality. This study considered complications in IAI patients diagnosed with Clavien-Dindo grade II.

### Definition of IAI

From postoperative hospitalization to the post-discharge outpatient follow-up period, physicians closely monitored the occurrence and progression of IAI in patients. According to the findings during the second operation, clinical symptoms, temperature ≥ 38°C (15), abdominal signs such as tenderness and rebound pain, laboratory tests such as leukocyte, CRP (C-reactive protein), and PCT (Procalcitonin) (15), culture results of drainage fluid, and abdominal CT (computerized tomography) were performed to check whether the patient had an intra-abdominal infection (16). IAI can be divided into two categories according to whether it is caused by intestinal leakage. The first category includes anastomotic and duodenal stump leakage, and the second category is abdominal effusion accompanied by infection without intestinal leakage. The anastomotic fistula was confirmed by endoscopy, laboratory examination, radiological examination (17), or secondary surgical exploration (18).

### Statistical analysis

Statistical analyses were performed using the IBM SPSS Statistics 25, R statistical software, and the Graphpad Prism 8 for Windows OS. The results of continuous variables were presented as the mean and standard deviation, and the categorical variables were presented as frequencies. The differences between groups for continuous variables were compared using an independent sample T-test, while the differences between groups for categorical variables were compared by the Chi-square test or Fisher’s exact test. The ROC (receiver operating characteristic) curve of each variable was drawn by SPSS data processing software, and the Jorden index was calculated to determine the critical value of each variable. The training data set used univariate and multivariate logistic regression analyses to screen risk factors and establish an RF model. In the univariate analysis, variables with P-value<0.1 were included in the multivariate logistic regression analysis (Forward: LR). P<0.05 were considered statistically significant in multivariate logistic regression analysis, and a nomogram was created using the R statistical software (R Foundation for Statistical Computing, Vienna, Austria). The score obtained can be converted into the probability of IAI occurrence prediction by substituting the data from the validation set into the equation obtained by binary logistic regression analysis. The ROC curve was applied to calculate the accuracy of the nomogram to predict the diagnosis of IAI. The model’s validity was measured using the AUROC (area under the receiver operating characteristic curve). A model with an AUROC above 0.7 was considered useful in diagnostic accuracy (19). The GraphPad Prism (Version 8) was used to describe the survival curves of the two groups.

## Results

### Incidence and clinical outcomes

The baseline characteristics of the training data set and validation data set were displayed in **Tables 1** and **2**. In 472 patients of the training data set who underwent surgical treatment (1. Radical gastrectomy with D2 lymph node dissection, 2. Palliative gastric cancer surgery) with primary gastric cancer, 34 (7.2%) patients suffered from intra-abdominal infection, including 15(44.1%) cases of anastomotic leakage or duodenal stump leakage, 19 (55.8%) cases of peritoneal effusion with infection. As demonstrated in **Tables 1** and **2**, the length of hospital stay in IAI patients was significantly longer in terms of short-term prognosis. Studies have revealed that intra-abdominal abscess is one of the common causes of readmission (20), severely affecting patients’ prognosis and quality of life. According to the Extended Clavien-Dindo classification of intra-abdominal infection (21), 1 case (2.9%) reached II, 30 cases (88.2%) reached IIIa, 3 cases (8.9%) reached IIIb stage. Fortunately, none of the patients had multiple organ failures or died from an intra-abdominal infection. Under the careful management of doctors and nurses in the treatment group, all the patients were improved and discharged after sufficient drainage, antibiotics, and other symptomatic support treatment (22).

**Table 1:** Clinicopathological characteristics of the patients in training data (n = 472).

Variable	IAI (n = 34)	Non-IAI (n = 438)	χ2 or *t-*value	*P*-value
Sex (Male: Female)	25:9	325:113	0.007	0.931
Age (years) #	66.26 ± 9.665	64.32 ± 11.109	0.991	0.322
BMI (kg/m2)#	23.19 ± 3.07	22.29 ± 3.05	1.648	0.100
Preoperative white blood cell count (×10^9/L) #	6.50 ± 1.95	6.14 ± 2.23	-0.891	0.373
Preoperative lymphocyte count (×10^9/L) #	1.55 ± 0.56	1.61 ± 0.59	-0.60	0.551
Preoperative hemoglobin (g/L) #	111.38 ± 21.68	117.39 ± 25.15	-1.354	0.177
Preoperative albumin (g/L) #	36.20 ± 4.84	38.00 ± 4.80	-2.104	0.036
PNI	43.95 ± 6.20	46.12 ± 6.29	-1.933	0.054
ASA (1 + 2/3+4)	30:4	425:13	4.726	0.03
Diabetes mellitus (yes/no)	3:31	47:391	0.003	0.953
Hypertension (yes/no)	19:15	119:319	12.573	< 0.001
History of abdominal surgery (yes/no)	8:26	45:393	4.311	0.038
Neoadjuvant chemotherapy (yes/no)	2:32	17:421	0.014	0.905
Lesion location (limited/diffuse)	33:1	435:3	0.000	1.000
Upper	5	64		
Middle	7	62		
lower	21	309		
other	1	3		
Time of operation (min)	205.91 ± 58.12	188.99 ± 46.81	1.993	0.047
Operation type				
Radical surgery: non-radical surgery	28:6	385:53	0.453	0.501
Operation method				
Open : Laparoscopic-assisted	25:9	347:91	0.613	0.434
Combined organ excision (yes/no)	6:28	20:418	8.011	0.005
BTF (yes/no)	9:25	73:365	2.113	0.146
Pathological type				
Signet-ring cell carcinoma: Non-signet ring cell carcinoma	3:31	89:349	2.657	0.103
Tumor stage (I+II/III+IV)	11:23	215:223	3.540	0.060
I	5	119		
II	6	96		
III	15	149		
IV	8	74		
Post-operative hospital stays (days) #	27.06 ± 14.043	14.30 ± 6.392	5.257	< 0.001

ASA, American Society of Anesthesiologist; BMI, Body Mass Index; #mean ± SD, IAI, intra-abdominal infection.

**Table 2:** Clinicopathological characteristics of the patients in internal validation data (n = 135).

Variable	IAI (n = 15)	Non-IAI (n = 120)	χ2 or *t-*value	*P*-value
Sex (Male: Female)	11:4	76:44	0.582	0.446
Age (years) #	69.40 ± 8.175	64.76 ± 10.971	-1.582	0.116
BMI (kg/m2)#	22.42 ± 3.08	23.06 ± 2.83	0.810	0.419
Preoperative white blood cell count (×10^9/L) #	6.46 ± 2.12	5.98 ± 1.85	-0.917	0.361
Preoperative lymphocyte count (×10^9/L) #	1.41 ± 0.55	1.57 ± 0.53	1.089	0.278
Preoperative hemoglobin (g/L) #	117.40 ± 15.33	116.73 ± 21.93	-0.114	0.909
Preoperative albumin (g/L) #	36.11 ± 2.92	36.74 ± 3.98	0.588	0.557
PNI	43.19 ± 4.34	44.60 ± 5.17	1.016	0.311
ASA (1 + 2/3+4)	14:1	103:17	0.162	0.687
Diabetes mellitus (yes/no)	1:14	23:97	0.698	0.403
Hypertension (yes/no)	5:10	26:94	0.472	0.492
History of abdominal surgery (yes/no)	6:9	14:106	6.385	0.012
Neoadjuvant chemotherapy (yes/no)	2:13	6:113	0.489	0.485
Lesion location (limited/diffuse)	12:3	113:7	2.109	0.146
upper	5	14		
middle	3	31		
lower	4	68		
other	3	7		
Time of operation (min)	235.73 ± 47.35	218.36 ± 55.18	-1.166	0.246
Operation type				
Radical surgery: non-radical surgery	12:3	109:11	0.720	0.396
Operation method				
Open : Laparoscopic-assisted	8:7	30:90	3.984	0.046
Combined organ excision (yes/no)	2:13	3:117	1.876	0.171
BTF (yes/no)	4:11	16:104	0.970	0.325
Pathological type				
Signet-ring cell carcinoma: Non-signet ring cell carcinoma	1:14	25:95	0.930	0.335
Tumor stage (I+II/III+IV)	6:9	80:40	4.101	0.043
I	3	54		
II	3	26		
III	6	36		
IV	3	4		
Post-operative hospital stays (days) #	16.07 ± 7.94	11.79 ± 4.53	-3.124	0.002

ASA, American Society of Anesthesiologist; BMI, Body Mass Index; #mean ± SD, IAI, intra-abdominal infection.

**Figure 2:**
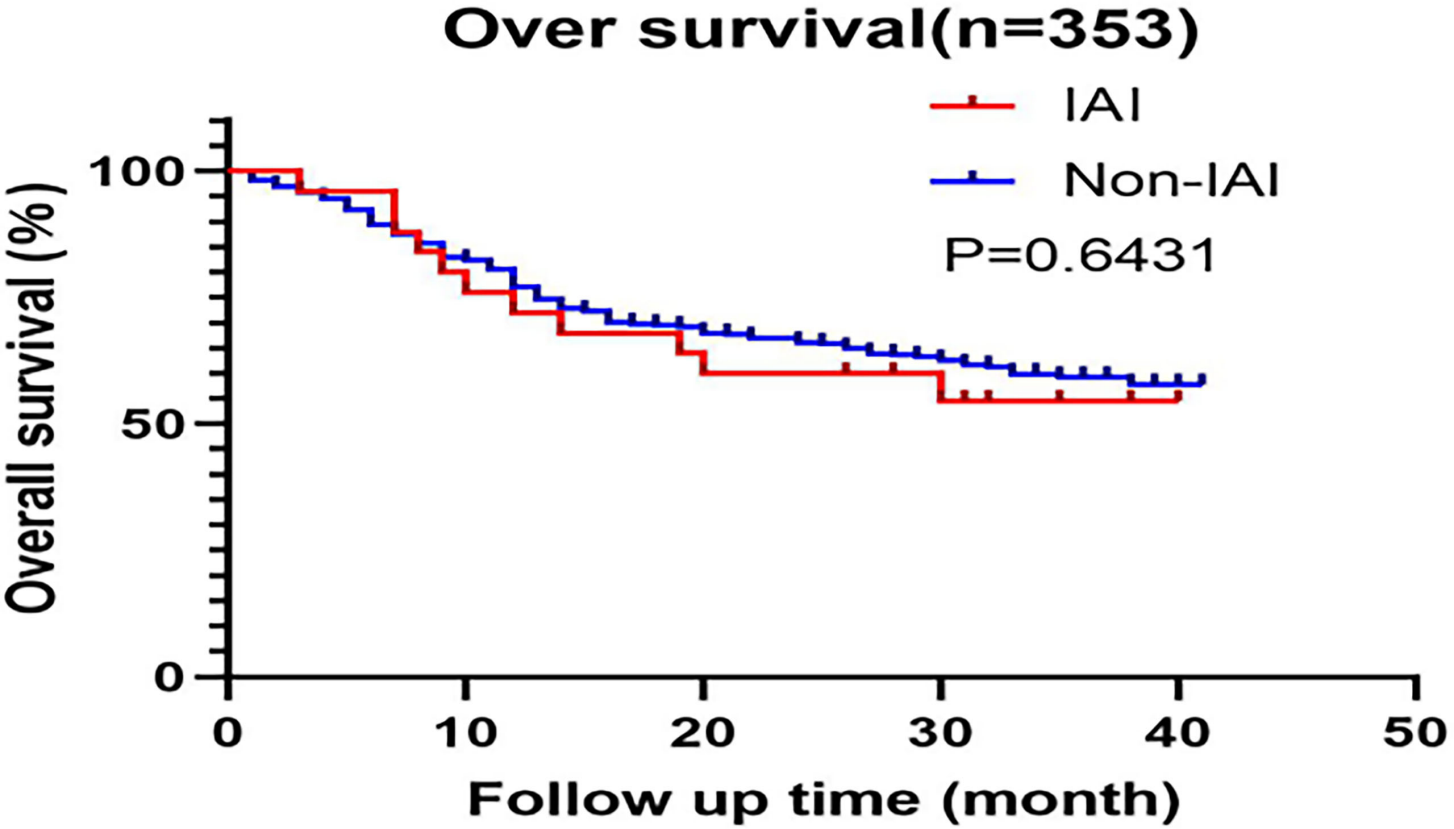
Kaplan-Meier curves of overall survival based on the training data.

The enrolled patients were contacted by phone to obtain and analyze their prognosis, with the most recent follow-up in May 2019. However, 25% of the patients were lost to follow-up and were excluded from the analysis. Finally, survival analysis was described in 353 patients with radical D1 or D2 lymphadenectomy. None of these patients died within 30 days after surgery. As is displayed in **Figure 2**, a significant difference did not appear in OS (overall survival) in the two groups (P=0.64). A study by Ru-Hong Tu et al. also demonstrated that intra-abdominal infection after therapeutic gastrectomy did not lead to reduced long-term survival in patients (23). Furthermore, neither overall nor major surgical complications were risk factors for decreased survival in patients who did not die from early postoperative complications within 30 days of surgery (24). That was also consistent with our research results.

### Pathogens

The abdominal drainage fluid of 34 patients diagnosed with IAI was cultured, and 19 (55.8%) were positive. The collection of abdominal drainage fluid follows the Sterile principle. Among the 19 patients with positive culture results, 4 had mixed growth of more than three strains (the possibility of specimen contamination could not be ruled out), 5 had mixed growth of two strains, and 10 had single strains. There were 6 gram-negative strains (46.2%), 6 gram-positive strains (46.2%), and 1 *Candida* spp. (7.6%). The most common microorganism was *Streptococcus anginosus*, *Enterococcus faecalis*, and *Klebsiella pneumoniae*. *Streptococcus anginosus* is one of the common colonized bacteria of the oropharynx, which can migrate to the digestive tract and become a pathogenic bacteria of postoperative intra-abdominal infection (25, 26). Previously, Xiao et al. (27) reported the presence of gram-negative bacilli in 73/1835, i.e., 4% of patients undergoing gastrectomy for gastric cancer. *Klebsiella pneumonia* was commonly linked to body mass index >25 kg/m2.

Due to the significant difference in pH (Pondus Hydrogenii) between the oral cavity and the stomach, it is generally believed that the bacteria do not remain active during the migration process in the digestive tract. Therefore, there are two possibilities: first is that for gastric cancer patients, a measure of perioperative management is using proton pump inhibitors, which can reduce the pH of the gastric mucosa. The second is that the most common site of gastric cancer is the antrum, so the site secreting more gastric acid was just removed during the operation.

### Risk factors

According to the univariate analysis of this data (**Table 3**), the IAI would occur easier in patients with a BMI ≥ 25 kg/m2, ASA score ≥ 3, history of abdominal surgery, hypertension, combined organ excision, and operative time ≥ 240 min. The tumor stage (III + IV) was a potential risk factor. Diabetes, radical surgery, or laparoscopic-assisted surgery execution, in addition to the pathological type and tumor stage, were not considered risk factors for the occurrence of IAI. The multivariate analysis demonstrated that hypertension (Odds Ratio [OR] = 3.408, 95% confidence interval [CI]: 1.632–7.117, P = 0.001), operation time ≥ 240 min (OR = 3.091, 95% CI: 1.408–6.783, P = 0.005), the history of abdominal surgery (OR = 2.609, 95% CI: 1.042–6.530, P = 0.041), and combined organ resection (OR = 4.123, 95% CI: 1.403–12.121, P = 0.01) were independent risk factors for IAI (**Table 4**).

**Table 3:** Univariate analysis of possible predictors of risk of IAI based on training data.

Variable	IAI (n = 34)	Non-IAI (n = 438)	Univariate analysis		
			OR	95%CI	*P*-value
Sex (Male: Female)	25:9	325:113	0.968	0.465-2.016	0.931
Age (years) ≥65/<65	22:12	236:202	1.521	0.771-3.001	0.222
BMI (kg/m2) ≥25/<25	12:22	87:351	2.055	1.054-4.006	0.033
Preoperative white blood cell count (×10^9/L) ≥4/<4	33:1	398:40	3.139	0.441-22.364	0.358
Preoperative lymphocyte count (×10^9/L)<0.8/≥0.8	33:1	21:417	1.806	0.594-5.490	0.532
Preoperative hemoglobin (g/L)<100/≥100	10:24	102:336	1.339	0.661-2.715	0.419
Preoperative albumin (g/L)<35/≥35	10:24	97:341	1.421	0.702-2.878	0.330
PNI<47/≥47	24:10	238:200	1.924	0.941-3.932	0.066
ASA (3 + 4/1+2)	4:30	13:425	3.569	1.416-8.992	0.03
Diabetes mellitus (yes/no)	3:31	47:391	0.817	0.259-2.575	0.953
Hypertension (yes/no)	19:15	119:319	3.066	1.605-5.856	0.000
History of abdominal surgery (yes/no)	8:26	45:393	2.433	1.162-5.094	0.038
Neoadjuvant chemotherapy (yes/no)	2:32	17:421	1.490	0.385-5.764	0.905
Time of operation (min) ≥240/<240	13:21	64:374	3.176	1.663-6.066	< 0.001
Operation type	28:6	385:53	0.667	0.288-1.542	0.501
Radical surgery: non-radical surgery					
Operation method	25:9	347:91	0.747	0.360-1.548	0.434
Open : Laparoscopic-assisted					
Combined organ excision (yes/no)	6:28	20:418	3.676	1.671-8.084	0.005
BTF (yes/no)	9:25	73:365	1.712	0.830-3.531	0.146
Pathological type	3:31	89:349	0.400	0.125-1.279	0.103
Signet-ring cell carcinoma: Non-signet ring cell carcinoma					
Tumor stage (I+II/III+IV)	11:23	215:223	1.921	0.958-3.851	0.060

**Table 4:** Multivariate analysis of risk factors of IAI based on internal validation data.

Risk factors	β coefficients	Standard error (SE)	Odds Ratio [OR]	95% Confidence Interval [CI]	P value
Intercept	-3.63	0.327			< 0.001
Hypertension	1.226	0.376	3.408	1.632-7.117	0.001
History of abdominal surgery	0.959	0.468	2.609	1.042-6.53	0.041
Operation time (min): ≥240	1.128	0.401	3.091	1.408-6.783	0.005
Combined organ excision	1.417	0.550	4.123	1.403-12.121	0.010

### Regression function model and validation

**Figure 3:**
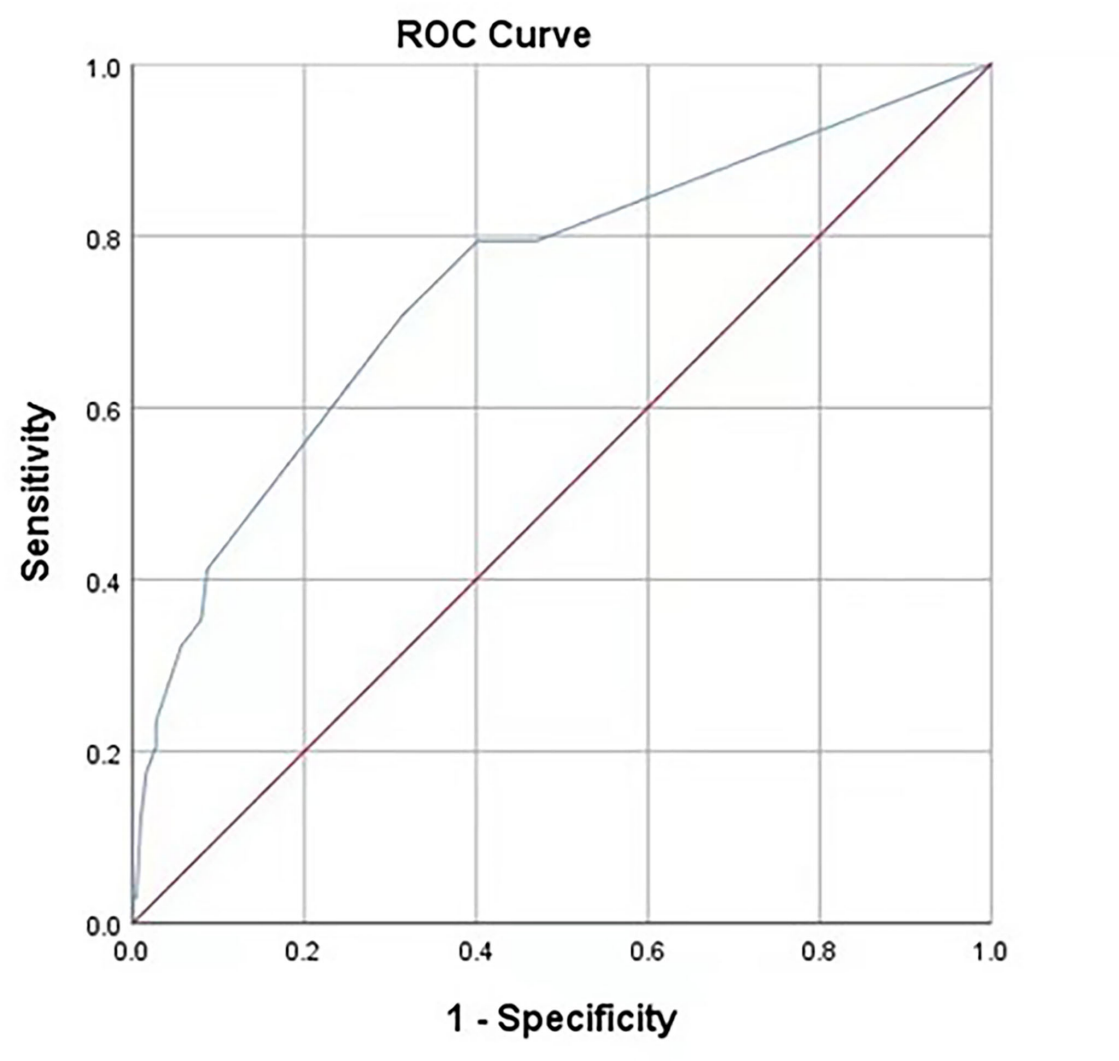
Receiver operating characteristic curves of the predictive model on the training data. AUC (95%CI) = 0.745 (0.650-0.840). The areas under receiver operating characteristic curves were 0.745±0.048 (P < 0.001). The ideal area under the curve was 1.00. The reference line represents that based on chance alone (area under the curve 0.50).

**Figure 4:**
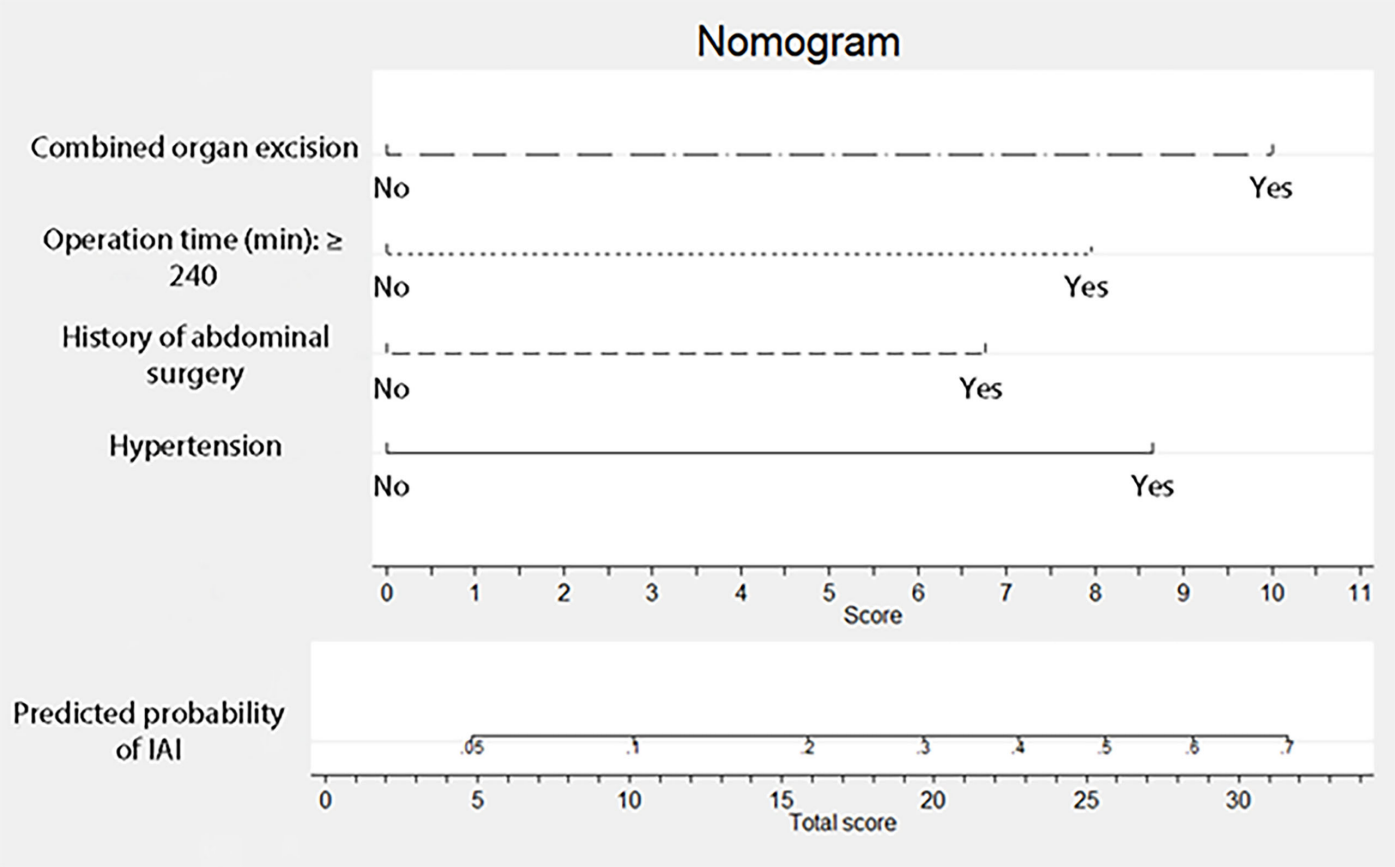
Nomogram for intra-abdominal infection after surgery for gastric cancer. To estimate the probability of intra-abdominal infection, mark patient values at each axis, draw a straight line perpendicular to the point axis, and sum the points for all variables. Next, mark the sum on the total point axis and draw a straight line perpendicular to the probability axis.

**Figure 5:**
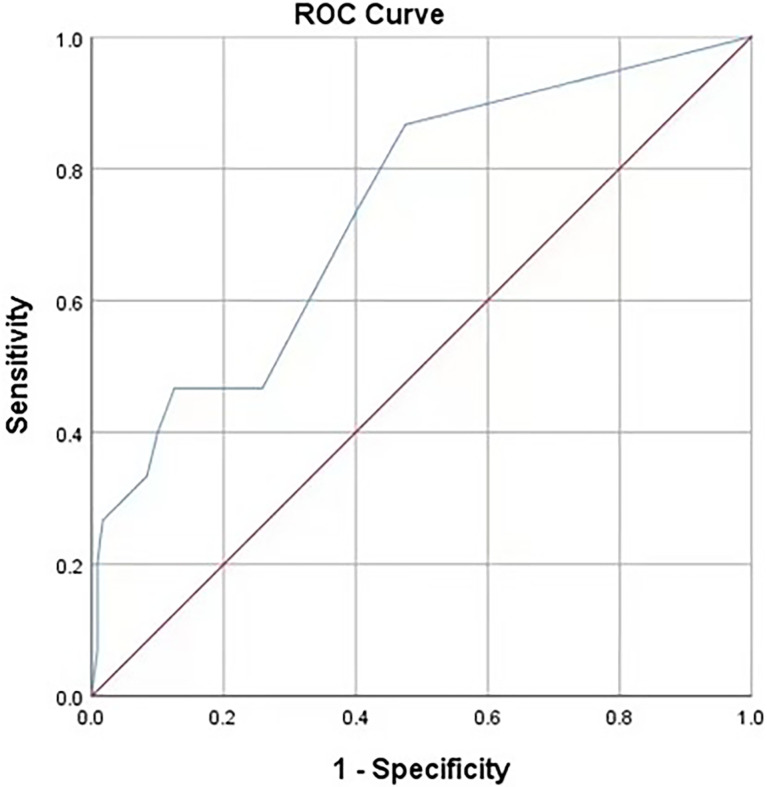
Receiver operating characteristic curves of the predictive model based on the internal validation data set. AUC (95%CI) = 0.736 (0.602-0.871), P=0.003.

According to the analysis results in **Table 4**, the RF model for IAI could be obtained as follows: $estimated\;probability=\frac{1}{1+EXP(-x)}$ , X=−3.63+(1.226*hypertension)+(0.959*history of abdominal surgery)+(1.128*operation time≥240mins)+(1.417*combined organ excision) . The ROC curve for the RF model based on the training data set for the prediction of IAI is demonstrated in **Figure 3** (AUROC=0.745 ± 0.048, P<0.001, 95% CI: 0.650–0.840). Intuitively, the RF model was presented as a nomogram that could visualize the RF model (**Figure 4**). The ROC curve of the nomogram based on the validation data set for the prediction of IAI is displayed in **Figure 5** (AUROC=0.736 ± 0.069, P=0.003, 95% CI: 0.602–0.871).

## Discussion

Gastric cancer still has a relatively high incidence, and surgical resection is the primary treatment method. Therefore, postoperative complications are important problems that front-line clinical workers should pay special attention to. Abdominal infection is one of gastric cancer’s most severe postoperative complications, resulting in significantly longer hospital stays, septic shock, multiple organ failures, and even death. In this single-center retrospective study, the incidence of postoperative abdominal infection for gastric cancer was 7.2%. A study by Felipe J.F.Coimbra MD (28) revealed that the overall incidence of postoperative complications of gastric cancer was 33.5%, among which the most common surgical complication was intra-abdominal abscess with an incidence of 7.9%, which was close to the data obtained in this retrospective study.

Chen Ke et al. have demonstrated that total laparoscopic gastrectomy has less bleeding, shorter hospitalization, and fewer postoperative complications than open gastrectomy (29). However, Inokuchi, M et al. ‘s meta-analysis demonstrated an insignificant difference in the intra-abdominal abscesses between the laparoscopic-assisted distal gastrectomy group and the open distal gastrectomy group (30). The results of this study also indicated that laparoscopic-assisted gastrectomy did not reduce the incidence of intra-abdominal infection.

Studies on high BMI (≥ 25 kg/m2) as a potential risk factor have drawn different conclusions. The meta-analysis by Zhao et al. demonstrated that high BMI patients had a higher risk of wound infection and IAI in both open and laparoscopic-assisted gastrectomy (31). However, the analysis by Sun et al. revealed that although high BMI patients had a higher risk of wound infection than those with low BMI (< 25 kg/m2), there was an insignificant difference in the incidence of anastomotic fistula among them (32). Previous studies have concluded that low PNI (< 47) is an independent risk factor for postoperative complications in patients with gastric cancer and will affect long-term prognosis (33). Therefore, it may be more useful than BMI in predicting postoperative IAI for gastric cancer patients. Our data analysis suggests that the low PNI (P=0.066) group may be at greater risk of developing IAI as a postoperative complication. The differences in the results of these studies may be due to sampling error. In addition, regional climate and dietary habits may make the BMI or PNI of a certain group generally higher or lower.

According to previous literature reports and clinicians’ experience, diabetes patients are more likely to develop infectious complications. It may be because of the physiological mechanisms, including lipid metabolism disorders, endothelial cell damage and dysfunction, abnormal platelet function, and blood vessel atherosclerosis, resulting in poor blood supply at the anastomotic and residual ends, thus increasing the risk of fistula (34). In addition, high blood pressure and diabetes often co-exist, causing damage to blood vessels together (35). Jönsson et al. (36) indicated that collagen synthesis depends on tissue oxygenation, thus demonstrating disturbed anastomotic healing in insufficient blood supply. This study found insignificant differences between the two groups, whether or not they had diabetes. Patients with hypertension, however, were at greater risk of developing IAI. It may be due to sampling error or bias.

Postoperative adhesions form in 50% to 100% of all abdominopelvic interventions (37). Due to the presence of more or less tissue adhesion in the abdominal cavity, patients with previous abdominal surgery must have separated adhesion next time. Then, the operation time will be prolonged.

Splenectomy and partial pancreas resection accounted for most of the combined organ resection. Spleen is the largest immune organ in the body, and its removal may affect the immune function of the human body. For example, splenectomy increases the risk of developing sepsis in response to *Streptococcus pneumoniae*, *Neisseria meningitides*, and *Hemophilus influenza* type B infections (38–40). There is also an increased risk of pancreatic fistula associated with infection in patients with partial pancreatectomy.

Patients with combined organ resection and a history of abdominal surgery generally have longer surgery times and longer gastrointestinal opening times, which increases the risk of surgical site infection.

The area under the receiver operating characteristic (ROC) curve for the RF model based on the training data set was 0.745 ± 0.048, and that of the nomogram based on the validation data set was 0.736 ± 0.069. It revealed that this nomogram had good predictive power.

IAI is one of the common complications of abdominal surgery, which can be life-threatening to a certain extent and cannot be ignored. Therefore, it is urgent to thoroughly study the risk factors of abdominal infection and its influence on the prognosis to better guide clinical work. This retrospective analysis demonstrated that hypertension, combined organ resection, history of abdominal surgery, and operation time ≥ 240 min were independent risk factors that could increase the risk of postoperative intra-abdominal infection. Therefore, we should minimize unnecessary tissue damage to reduce the wound surface and the operation time. Stijn Blot et al. summarized the etiological characteristics of 1,982 patients with intra-abdominal infection. They found that most patients were infected with gram-negative bacteria, among which *Escherichia coli* in Enterobacteria was the most common, and *Enterococcus* sp. accounted for the most in gram-positive bacteria (41). Our data demonstrated that the top three pathogens were *Streptococcus anginosus*, *Klebsiella pneumoniae*, and *Enterococcus faecalis*. *Streptococcus anginosus* is a commonly colonized bacteria in the oral cavity. K*lebsiella pneumoniae* and *Enterococcus faecalis* are common colonized bacteria in the intestinal tract. From the standpoint of pathogens, we must conduct well in perioperative oral management. Patients with severe periodontitis need to be treated by a stomatologist before surgery. Moreover, it is necessary to make good intestinal preparation before operation (42), strictly follow the principle of sterility during operation, apply a sufficient course of antibiotics after the operation, and ensure a good drainage effect.

## Conclusions

IAI is allied with gastric cancer surgery complications, including pathogenic growth. Species such as *Klebsiella, Streptococcus*, and *Enterococcus* dominated the variety in this study. Independent risk factors impacting IAI included hypertension, combined organ resection, history of abdominal surgery, and operation time of more than 240 mins. Diabetes did not increase the chance of infection. Compared to conventional electrosurgery, the extent of operative time may be reduced with energy devices, such as ultrasonically activated coagulating shears. Since this study is a single-center retrospective study, there is a possibility that the samples taken do not conform to the general population. Besides, the selective and observational bias in the retrospective study are also limitations of this type of study. A larger sample size and patients from diverse areas could help reduce these limitations. In conclusion, gastric cancer patients with the risk factors above require more attention. This is the first study to establish an RF model of IAI and verify it, and the verified result shows that the RF model has a significant predictive ability for the occurrence of IAI after gastric cancer surgery.

## Data availability statement

The data supporting the findings are included in the article/supplementary material. Further inquiries can be directed to the corresponding authors.

## Author contributions

YZ, ZW, XC, and WH designed this study. YZ collected and analyzed the data, made tables and figures, and wrote the manuscript. ZB, MH, XC, and WH revised the manuscript. All authors contributed to the article and approved the submitted version.

## Conflict of interest

The authors declare that the research was conducted in the absence of any commercial or financial relationships that could be construed as a potential conflict of interest.

## Publisher’s note

All claims expressed in this article are solely those of the authors and do not necessarily represent those of their affiliated organizations, or those of the publisher, the editors and the reviewers. Any product that may be evaluated in this article, or claim that may be made by its manufacturer, is not guaranteed or endorsed by the publisher.

## References

[1] Sung H Ferlay J Siegel RL Laversanne M Soerjomataram I Jemal A. Global cancer statistics 2020: GLOBOCAN estimates of incidence and mortality worldwide for 36 cancers in 185 countries. CA Cancer J Clin (2021) 71(3):209–49. doi: 10.3322/caac.21660 33538338

[2] Yuan Y. A survey and evaluation of population-based screening for gastric cancer. Cancer Biol Med (2013) 10(2):72–80. doi: 10.7497/j.issn.2095-3941.2013.02.002 23882421 PMC3719193

[3] Ono H Yao K Fujishiro M Oda I Nimura S Yahagi N. Guidelines for endoscopic submucosal dissection and endoscopic mucosal resection for early gastric cancer. Dig Endosc. (2016) 28(1):3–15. doi: 10.1111/den.12518 26234303

[4] Tokunaga M Tanizawa Y Bando E Kawamura T Terashima M. Poor survival rate in patients with postoperative intra-abdominal infectious complications following curative gastrectomy for gastric cancer. Ann Surg Oncol (2013) 20(5):1575–83. doi: 10.1245/s10434-012-2720-9 23076557

[5] Sano T Sasako M Yamamoto S Nashimoto A Kurita A Hiratsuka M. Gastric cancer surgery: morbidity and mortality results from a prospective randomized controlled trial comparing D2 and extended para-aortic lymphadenectomy–Japan clinical oncology group study 9501. J Clin Oncol (2004) 22(14):2767–73. doi: 10.1200/JCO.2004.10.184 15199090

[6] Papenfuss WA Kukar M Oxenberg J Attwood K Nurkin S Malhotra U. Morbidity and mortality associated with gastrectomy for gastric cancer. Ann Surg Oncol (2014) 21(9):3008–14. doi: 10.1245/s10434-014-3664-z 24700300

[7] Kim EH Park JC Song IJ Kim YJ Joh DH Hahn KY. Prediction model for non-curative resection of endoscopic submucosal dissection in patients with early gastric cancer. Gastrointest Endosc (2017) 85(5):976–83. doi: 10.1016/j.gie.2016.10.018 27756614

[8] Wu L Ge L Qin Y Huang M Chen J Yang Y. Postoperative morbidity and mortality after neoadjuvant chemotherapy versus upfront surgery for locally advanced gastric cancer: a propensity score matching analysis. Cancer Manag Res (2019) 11:6011–8. doi: 10.2147/CMAR.S203880 PMC6614824 31308742

[9] Zhou Y Tian Z Zeng J Zhou W Wu K Shen W. Effect of neoadjuvant treatment combined with radical gastrectomy on postoperative complications and prognosis of gastric cancer patients. Scand J Gastroenterol (2021) 56(11):1343–8. doi: 10.1080/00365521.2021.1966092 34415219

[10] Amin MB Greene FL Edge SB Compton CC Gershenwald JE Brookland RK. The eighth edition AJCC cancer staging manual: Continuing to build a bridge from a population-based to a more "personalized" approach to cancer staging. CA Cancer J Clin (2017) 67(2):93–9. doi: 10.3322/caac.21388 28094848

[11] Japanese Gastric Cancer Association Japanese gastric cancer treatment guidelines 2014 (ver. 4). Gastric Cancer (2017) 20(1):1–19. doi: 10.1007/s10120-016-0622-4 PMC5215069 27342689

[12] Sartelli M Chichom-Mefire A Labricciosa FM Hardcastle T Abu-Zidan FM Adesunkanmi AK. The management of intra-abdominal infections from a global perspective: 2017 WSES guidelines for management of intra-abdominal infections. World J Emerg Surg (2017) 12:29. doi: 10.1186/s13017-017-0141-6 28702076 PMC5504840

[13] Onodera T Goseki N Kosaki G. [Prognostic nutritional index in gastrointestinal surgery of malnourished cancer patients]. Nihon Geka Gakkai Zasshi (1984) 85(9):1001–5. 6438478

[14] Xiao H Quan H Pan S Yin B Luo W Huang G. Impact of peri-operative blood transfusion on post-operative infections after radical gastrectomy for gastric cancer: a propensity score matching analysis focusing on the timing, amount of transfusion and role of leukocyte depletion. J Cancer Res Clin Oncol (2018) 144(6):1143–54. doi: 10.1007/s00432-018-2630-8 PMC5948291 29572591

[15] Vincenzi B Fioroni I Pantano F Angeletti S Dicuonzo G Zoccoli A. Procalcitonin as diagnostic marker of infection in solid tumors patients with fever. Sci Rep (2016) 6:28090. doi: 10.1038/srep28090 27312877 PMC4911581

[16] Emmi V Sganga G. Clinical diagnosis of intra-abdominal infections. J Chemother (2009) 21 Suppl 1:12–8. doi: 10.1179/joc.2009.21.Supplement-1.12 19622446

[17] Haaga JR. Imaging intraabdominal abscesses and nonoperative drainage procedures. World J Surg (1990) 14(2):204–9. doi: 10.1007/BF01664874 2183483

[18] Facy O Paquette B Orry D Binquet C Masson D Bouvier A. Diagnostic accuracy of inflammatory markers as early predictors of infection after elective colorectal surgery: Results from the IMACORS study. Ann Surg (2016) 263(5):961–6. doi: 10.1097/SLA.0000000000001303 26135691

[19] Giannini EG Zaman A Kreil A Floreani A Dulbecco P Testa E. Platelet count/spleen diameter ratio for the noninvasive diagnosis of esophageal varices: results of a multicenter, prospective, validation study. Am J Gastroenterol (2006) 101(11):2511–9. doi: 10.1111/j.1572-0241.2006.00874.x 17029607

[20] Asaoka R Kawamura T Makuuchi R Irino T Tanizawa Y Bando E. Risk factors for 30-day hospital readmission after radical gastrectomy: a single-center retrospective study. Gastric Cancer (2019) 22(2):413–20. doi: 10.1007/s10120-018-0856-4 30006830

[21] Katayama H Kurokawa Y Nakamura K Ito H Kanemitsu Y Masuda N. Extended clavien-dindo classification of surgical complications: Japan clinical oncology group postoperative complications criteria. Surg Today (2016) 46(6):668–85. doi: 10.1007/s00595-015-1236-x PMC4848327 26289837

[22] Solomkin JS Mazuski JE Bradley JS Rodvold KA Goldstein EJ Baron EJ. Diagnosis and management of complicated intra-abdominal infection in adults and children: guidelines by the surgical infection society and the infectious diseases society of America. Clin Infect Dis (2010) 50(2):133–64. doi: 10.1086/649554 20034345

[23] Tu RH Lin JX Desiderio J Li P Xie JW Wang JB. Does intra-abdominal infection after curative gastrectomy affect patients' long-term prognosis? a multi-center study based on a Large sample size. Surg Infect (Larchmt) (2019) 20(4):271–7. doi: 10.1089/sur.2018.246 30720387

[24] Galata C Blank S Weiss C Ronellenfitsch U Reissfelder C Hardt J. Role of postoperative complications in overall survival after radical resection for gastric cancer: A retrospective single-center analysis of 1107 patients. Cancers (Basel) (2019) 11(12):1980. doi: 10.3390/cancers11121890 PMC6966624 31783704

[25] Sasaki M Kodama Y Shimoyama Y Ishikawa T Kimura S. Aciduricity and acid tolerance mechanisms of streptococcus anginosus. J Gen Appl Microbiol (2018) 64(4):174–9. doi: 10.2323/jgam.2017.11.005 29669961

[26] Nishikawa M Honda M Kimura R Kobayashi A Yamaguchi Y Hori S. The bacterial association with oral cavity and intra-abdominal abscess after gastrectomy. PloS One (2020) 15(11):e0242091. doi: 10.1371/journal.pone.0242091 33166362 PMC7652288

[27] Xiao H Xiao Y Quan H Liu W Pan S Ouyang Y. Intra-abdominal infection after radical gastrectomy for gastric cancer: Incidence, pathogens, risk factors and outcomes. Int J Surg (2017) 48:195–200. doi: 10.1016/j.ijsu.2017.07.081 28751223

[28] Coimbra FJF de Jesus VHF Franco CP Calsavara VF Ribeiro HSC Diniz AL. Predicting overall and major postoperative morbidity in gastric cancer patients. J Surg Oncol (2019) 120(8):1371–8. doi: 10.1002/jso.25743 31696512

[29] Chen K Pan Y Cai JQ Xu XW Wu D Mou YP. Totally laparoscopic gastrectomy for gastric cancer: a systematic review and meta-analysis of outcomes compared with open surgery. World J Gastroenterol (2014) 20(42):15867–78. doi: 10.3748/wjg.v20.i42.15867 PMC4229555 25400474

[30] Inokuchi M Sugita H Otsuki S Sato Y Nakagawa M Kojima K. Laparoscopic distal gastrectomy reduced surgical site infection as compared with open distal gastrectomy for gastric cancer in a meta-analysis of both randomized controlled and case-controlled studies. Int J Surg (2015) 15:61–7. doi: 10.1016/j.ijsu.2015.01.030 25644544

[31] Zhao B Zhang J Mei D Luo R Lu H Xu H. Does high body mass index negatively affect the surgical outcome and long-term survival of gastric cancer patients who underwent gastrectomy: A systematic review and meta-analysis. Eur J Surg Oncol (2018) 44(12):1971–81. doi: 10.1016/j.ejso.2018.09.007 30348605

[32] Sun L Zhao B Huang Y Lu H Luo R Huang B. Feasibility of laparoscopy gastrectomy for gastric cancer in the patients with high body mass index: A systematic review and meta-analysis. Asian J Surg (2020) 43(1):69–77. doi: 10.1016/j.asjsur.2019.03.017 31036475

[33] Jiang N Deng JY Ding XW Ke B Liu N Zhang RP. Prognostic nutritional index predicts postoperative complications and long-term outcomes of gastric cancer. World J Gastroenterol (2014) 20(30):10537–44. doi: 10.3748/wjg.v20.i30.10537 PMC4130864 25132773

[34] Zawada AE Moszak M Skrzypczak D Grzymisławski M. Gastrointestinal complications in patients with diabetes mellitus. Adv Clin Exp Med (2018) 27(4):567–72. doi: 10.17219/acem/67961 29533548

[35] Yamazaki D Hitomi H Nishiyama A. Hypertension with diabetes mellitus complications. Hypertens Res (2018) 41(3):147–56. doi: 10.1038/s41440-017-0008-y 29353881

[36] Jönsson K Jiborn H Zederfeldt B. Breaking strength of small intestinal anastomoses. Am J Surg (1983) 145(6):800–3. doi: 10.1016/0002-9610(83)90144-7 6859418

[37] diZerega GS. Contemporary adhesion prevention. Fertil Steril (1994) 61(2):219–35. doi: 10.1016/S0015-0282(16)56507-8 8299773

[38] Amlot PL Hayes AE. Impaired human antibody response to the thymus-independent antigen, DNP-ficoll, after splenectomy. implications for post-splenectomy infections. Lancet (1985) 1(8436):1008–11. doi: 10.1016/S0140-6736(85)91613-7 2859463

[39] Ram S Lewis LA Rice PA. Infections of people with complement deficiencies and patients who have undergone splenectomy. Clin Microbiol Rev (2010) 23(4):740–80. doi: 10.1128/CMR.00048-09 PMC2952982 20930072

[40] Robinette CD Fraumeni JF Jr. Splenectomy and subsequent mortality in veterans of the 1939-45 war. Lancet (1977) 2(8029):127–9. doi: 10.1016/S0140-6736(77)90132-5 69206

[41] Blot S Antonelli M Arvaniti K Blot K Creagh-Brown B de Lange D. Epidemiology of intra-abdominal infection and sepsis in critically ill patients: "AbSeS", a multinational observational cohort study and ESICM trials group project. Intensive Care Med (2019) 45(12):1703–17. doi: 10.1007/s00134-019-05819-3 PMC6863788 31664501

[42] Toh JWT Phan K Hitos K Pathma-Nathan N El-Khoury T Richardson AJ. Association of mechanical bowel preparation and oral antibiotics before elective colorectal surgery with surgical site infection: A network meta-analysis. JAMA Netw Open (2018) 1(6):e183226. doi: 10.1001/jamanetworkopen.2018.3226 30646234 PMC6324461

